# Antibiotic Therapy and Vaginal Microbiota Transplantation Reduce Endometriosis Disease Progression in Female Mice *via* NF-κB Signaling Pathway

**DOI:** 10.3389/fmed.2022.831115

**Published:** 2022-03-30

**Authors:** Feilei Lu, Jing Wei, Yanying Zhong, Ying Feng, Bo Ma, Yifei Xiong, Kehong Wei, Buzhen Tan, Tingtao Chen

**Affiliations:** ^1^Department of Obstetrics and Gynecology, The Second Affiliated Hospital of Nanchang University, Nanchang, China; ^2^Institute of Translational Medicine, Nanchang University, Nanchang, China; ^3^Queen Mary School, Nanchang University, Nanchang, China

**Keywords:** endometriosis, pathogenesis, vaginal microbiota, NF-κB signaling pathway, high-throughput sequencing

## Abstract

Endometriosis (EMS) is a disease characterized by estrogen-dependent, chronic inflammatory, and annoying symptoms, which inflicts about 10% reproductive-age women. The diagnosis of endometriosis mainly depends on pathological examination after surgical resection while the pathogenesis of EMS is not clear enough. Surgical resection and drug therapy (including painkillers and hormone therapy, especially gonadotropin-releasing hormone analogs, GnRH-a) are widely used, but they are expensive and have many side effects. There are few studies on vaginal microorganisms in women with endometriosis. We collected vaginal secretions from women with EMS confirmed by pathology and demonstrated that they were different from that of healthy women by 16s rRNA high-throughput sequencing. Additionally, we established the EMS model in female mice by intraperitoneally injecting fragments from donor mice (3-week growth). Then, the mice were treated with mixed antibiotics (vagina) and NF-κB signaling pathway inhibitors (intraperitoneal injection), respectively. The result suggested that the ectopic lesions were inhibited. In addition, inflammatory cytokines IL-1β, IL-6, and TNF-α in peritoneal fluid, cell proliferation marker ki-67, and macrophage marker Iba-1 in ectopic lesions decreased significantly from that of mock mice. We also observed similar results as above by vaginal microbiota transplantation (VMT) and subcutaneous injection of leuprorelin acetate (LA, one of GnRH-a) for mice with EMS. These results showed that vaginal use of antibiotics or VMT is helpful to treat endometriosis in mice. However, due to the great difference between human and mouse vaginal microbiota, its mechanism and clinical transformation application still need to be further studied in the future.

## Introduction

Endometriosis, defined as the existence of endometrial-like tissue (stroma and glands) outside the uterine cavity, is an autoimmune disease harassing 6–10% women of childbearing age (170 million worldwide) and could lead to lower abdominal pain, dysmenorrhea, infertility, and abnormal menstruation (1–4). At present, the major therapeutic approaches for endometriosis include surgical resection of endometriotic lesions, non-steroidal anti-inflammatory drugs (NSAIDs), and hormone-based drug therapy (5, 6). However, these therapeutic options also brought up numerous adverse reactions, such as nausea, hypoestrogenism, damage to normal endometrial mucosa, and also a high recurrence rate of 50% at 5-year follow-up (5, 7). This leads to severe social burden with the decline in the quality of life, depression, anxiety, fatigue, as well as impairment in social relationship and productivity (8, 9). Therefore, there is an urgent need for a better understanding of the pathogenesis and development of endometriosis, to develop new therapeutic options to deal with this poignant disease.

Till now, a number of theories have been put forward to elucidate the pathogenesis of endometriosis, and the immune and inflammatory theories were widely accepted. The theory deems that many kinds of serum autoantibodies (high levels of IgG, IgA, IgM, and anti-endometrial antibodies) and tissue damage, immune cell dysfunction (abnormal lymphocyte activation, particularly antigen-presenting cells presenting autoantigen to autoreactive T cells, increased levels of peritoneal neutrophils and macrophages, reduced cytotoxic function of NK cells, and aberrant numbers of T and B lymphocytes), lead to the failure in removing endometrial debris in the pelvic cavity, finally aid endometriotic cell implantation, growth, and angiogenesis, and proliferation (4, 10, 11). Additionally, the activation of neutrophils and macrophages causes the release of proinflammatory cytokines, such as interleukin (IL)-6 and endometriotic lesions vasculogenesis, which leads to the growth and survival of endometriotic lesions (12, 13). A majority of proteins involved in the proinflammatory environment of endometriosis are increased by nuclear factor-kappaB (NF-κB) and have been demonstrated to promote endometriotic cell proliferation through Toll-like receptor–myeloid differentiation factor 88 (TLR-MyD88) signaling pathway (14). During this process, the proinflammatory cytokines, such as lipopolysaccharide (LPS) from bacteria, bind to receptors (TLR) on the endometriotic cells and activate the canonical NF-κB signaling pathway by phosphorylation and ubiquitination of IκB (NF-κB inhibitory protein), which allows the p65/p50 dimers (inactivated by IκB previously) to be phosphorylated to p-p65/p50 (activated) and the subsequent binding to DNA. This could initiate a cascade of upregulation of target genes, such as IL-6, IL-1β, and macrophage migration inhibitory factor (MIF) (15).

The vagina is a lumen in which more than 300 bacterial species gather together (16), and these bacteria are closely connected with a variety of gynecological diseases, such as bacterial vaginosis (BV), sexually transmitted diseases (STDs), which may be alleviated or cured by restoring normal vaginal microbiota (17, 18). Therefore, normal vaginal microbiota is also a momentous constituent to maintain vaginal health besides estrogen. Similar to the previous reports that many diseases are closely related to the imbalance of microorganisms, endometriosis may also be related to disorder of vaginal microbiota (19–21). Vaginal microorganisms of patients with endometriosis were different from healthy women, with a decrease in beneficial bacteria *lactobacillus* and an increase in harmful bacteria *atopobium* (22, 23). Reconstructing the vaginal microbiota environment by VMT, which recovers the microbiota diversity and normal composition, provided a new possibility to these diseases. However, the study on the intervention using vaginal microbiota is limited, which underscores the need for further research in this area.

In this study, we confirmed the differences of vaginal microorganisms between patients with endometriosis and healthy women by 16s rRNA high-throughput sequencing, suggesting that the vaginal microbiota may be related to endometriotic lesion progression. We then developed a mouse model of endometriosis and evaluated the effect of vaginal microbiota on endometriosis by vaginal use antibiotics and VMT, further confirmed its efficacy in treatment of endometriosis, and explored its potential mechanisms, providing a new therapeutic strategy for endometriosis.

## Materials and Methods

### Clinical Studies

A total of 34 women (control, *n* = 18; endometriosis, *n* = 16) were enrolled from 30 September, 2020 to 31 March, 2021. The inclusion criteria included the following: (1) aged 18–55 years and regular menstrual cycle (28 ± 7 days), (2) confirmed by imaging and pathology after surgery, (3) no asexual life, no vaginal flushing and vaginal medication within 2 days, no cervical treatment within 7 days, and no antibiotic drugs within 30 days, and (4) no abnormal object in vaginal secretion examination; the white blood cell count and neutrophil ratio in the blood routine test were normal. The exclusion criteria included the following: (1) drug treatment history within 3 months before treatment (oral contraceptives, progesterone, and other drugs for the treatment of endometriosis and other diseases, excluding gonadotropin-releasing hormone analog), and probiotics (including health-care products), (2) patients with intrauterine devices insertion, (3) pregnancy or lactation, and (4) surgical history or other diseases, such as vaginal cancer, acute or severe vaginal symptoms that require treatment, vaginal infection or onset, combined with autoimmune diseases, systemic acute inflammation, and genital tract malformations.

### Animal Studies

All 110 mice (7-week-old female Balb/C) were purchased from SJA Laboratory Animal (Hunan, China). The mice were fed in the specific pathogen-free laboratory center under standard conditions (12-h light–12-h dark cycle).

Totally, 90 mice were randomly divided into donors (*n* = 30) and recipients (*n* = 60) and were used to establish the endometriosis model by intraperitoneal injection of endometrial segments (24, 25). Donors and recipients were injected subcutaneously with estradiol benzoate (3 μg/mouse, Huachu, Xinxiang, China). Then, 1 week after donor mice were sacrificed, their uteri were stripped immediately, placed in a Petri dish containing warm saline, and the endometrial tissues were isolated, whose maximal diameter was less than 1 mm. To avoid possible bias, the endometrium fragments from every 2 donors were mixed. Every 4 recipients have intraperitoneally injected the segments above with a 1-ml sterile syringe. Then, 3 weeks after endometrial tissue injection, ultrasonography examination was used to confirm the endometriotic lesions. Also, 2% isoflurane (H.F.Q BIO-TECHNOLOGY Co., Ltd., Jiangsu, China) in oxygen was used to anesthetize the mice. Then, the mice were fixed in the supine position on a flat stage. The depilatory paste and medical ultrasonic couplant (RuiTaiQi, Shandong, China) were used on the abdomen of mice. The Vevo 2100 high-resolution small animal ultrasound system (VisualSonics, FUJIFILM, America) and an MS-400 mouse electronic linear array probe (VisualSonics) were used to conduct imaging (26). Finally, 60 endometriotic mice were successfully constructed.

Totally, 10 mice were randomly divided into the C group, and 30 endometriosis mice were divided into the M group (the vaginas of mice were plugged in absorbable gel sponge with PBS, *n* = 10), the ABX group [the vaginas of mice were plugged in absorbable gel sponge with a mixed antibiotic solution of ABX (vancomycin 0.5 mg/ml, neomycin 1 mg/ml, metronidazole 1 mg/ml, and ampicillin 1 mg/ml) once every 3 days for 21 days, *n* = 10], and the parthenolide group [intraperitoneally injected parthenolide 4 times a week for 21 days (27), *n* = 10].

In another grouping scheme, 10 mice were divided into the C group, and 30 remaining endometriotic mice were divided into the M group (*n* = 10), the leuprorelin acetate group [LA microspheres sustained-release (Livzon Co., Ltd., Guangdong, China) was injected subcutaneously at abdomen once (28, 29), *n* = 10] and the VMT group (*n* = 10).

Finally, mice were euthanized and the peritoneal fluid was collected. Anatomical features of the endometrium and ectopic lesions were observed and the weight and volume were recorded.

### Vaginal Microbiota Transplantation Process

The vaginal secretions from healthy mice were collected and centrifuged at 10,000 rpm/min for 10 min. Then, they were washed two times with sterile PBS and the supernatant was discarded. After that, the vaginal microbiota was precipitated and suspended with an equal volume of sterile PBS. The absorbent gelatin sponge was cut into small squares and then placed in PBS suspension containing normal vaginal microbiota to make it fully contact with the bacterial solution. The sponge was inserted into the uterine cavity through the vagina with tweezers and repeated every 3 days (18, 30).

### Bacterial 16S rRNA Gene Sequencing and Diversity Analysis

Vaginal secretion from women was collected, and the bacterial genomic DNA was extracted using TIANamp Bacteria DNA Kit (TianGen) according to the manufacturer’s instructions. The concentration and quality of DNA were determined by NanoDrop spectrophotometer. The 16S ribosomal DNA (rDNA) V4 region was amplified using primers (F,AYTGGGYDTAAAGNG; R,TACNVGGGTATCTAATCC), and the PCR products were sequenced on the Illumina HiSeq 2000 platform (Illumina, Inc.). Amplicon generation and sequencing were performed by the Personalbio Co., Ltd. (Shanghai, China). Paired-end reads were processed to samples. QIIME software (version 2.0) was used to denoise and dereplication sequences to obtain high-quality tags. GreenGene Database was applied to note on species taxonomy. Sequences with ≥ 97% similarity were assigned to the same operational taxonomic units (OTUs). The dataset was used for downstream α diversities (indexes of Shannon, Simpson), β diversities (principle coordinates analysis, PCoA), and linear discriminant analysis (LDA). Sequences with FASTQ format were uploaded to the NCBI (accession number: PRJNA784739).

### Histological Analysis

Paraformaldehyde-fixed tissues were serially sectioned into 5-μm thick sections. Tissue sections were fixed, processed, embedded, dewaxing, and dyeing for hematoxylin and eosin staining. For immunohistochemical staining, sections were blocked and incubated overnight at 4°C in 2% goat serum with primary antibodies. The primary antibodies were purchased from Abcam, anti-Iba1 (ab178847) and anti-ki-67 (ab16667). Sections were then incubated with biotinylated secondary antibody.

### Western Blot Analysis

Uterine tissues were homogenized, and proteins were isolated using normal sodium. The purity of proteins was detected and then resolved using SDS-PAGE (polyacrylamide gel electrophoresis). The proteins were transferred to the polyvinylidene difluoride (PVDF) membranes and blocked with 5% bovine serum albumin (BSA) in Tris-buffered saline with Tween 20 for 1.5 h at room temperature. After that, primary antibodies and membranes were co-incubated overnight at 4°C, and the membranes were incubated with the secondary antibody conjugated with HRP. Antibodies against TLR-4, p-65, p-p-65, and GAPDH were purchased from Proteintech Group, Inc. (Wuhan, China). Antibodies against MyD88 (ab219413) were purchased from Abcam (San Francisco, CA, United States).

### RNA Preparation and q-PCR

TRIzol reagent (Gibco BRL, Grand Island, NY, United States) was used according to the fabricant’s instruction to isolate RNA of uterine tissues. After that, a NanoDrop 2000 spectrophotometer (Thermo Fisher Scientific, Waltham, MA, United States) was applied to detect the concentration of RNA. RNA was reverse-transcribed into cDNA using a commercially available kit (PrimeScript RT Master Mix; TaKaRa Biotechnology). Quantitative real-time PCR was conducted with a 7900 HT fast real-time PCR system (ABI, Foster City, CA, United States).

### Statistical Analysis

A two-tailed paired Student’s *t*-test was used for statistical significance testing for all data except the 16S rRNA sequencing data. GraphPad Software version 8.0 (San Diego, CA, United States) was used for statistical analysis. The non-parametric Kruskal–Wallis test was used to compare the Shannon diversities of microbiota. *p* < 0.05 was considered statistically significant.

## Results

### Clinical Indicators Comparison in Patients With Endometriosis and Healthy Women

A total of 16 patients with endometriosis and 18 healthy women of reproductive age were included in this study. The lesions of patients with endometriosis were rated according to the Revised American Society for Reproductive Medicine (r-ASRM) score, with 9 patients classified as stage 3–4 (advanced stage, score 16–40) and 7 patients classified as stage 1–2 (early stage, score 1–15). The diagnosis of all patients with endometriosis was made by pathological examination after the operation. Although no significant difference in age and body mass index (BMI) between the endometriosis group and control group was found, the abortions number in the endometriosis group is higher than that in the control group. As for blood test results, anti-Müllerian hormone (AMH) of endometriosis group, which represented ovarian reserve function, was less than that of control group. Estradiol (E2) in endometriosis group was significantly higher than control group for the hormone which is necessary for the growth of endometriosis. Carbohydrate antigen 125 (CA125), a glycoprotein found in sera from patients with ovarian cancer, breast cancer, and pancreatic cancer, had more abundant concentration in endometriosis group and may be an auxiliary index for diagnosing endometriosis. Neutrophil-to-lymphocyte ratio (NLR), the ratio of two kinds of cells in the blood that was used as an indicator of inflammation, was higher in the endometriosis group compared to the control group (Table 1).

**TABLE 1:** Basic characteristics of the included clinical study population.

	Endometriosis group (*n* = 16)	Control group (*n* = 18)	*P* value
Age (years)	36.75 ± 7.11	35 ± 6.61	0.4625
BMI (kg/m^2^)	20.64 ± 3.04	19.75 ± 1.47	0.2846
Abortions (number)	0.88 ± 0.96	0.11 ± 0.32	<0.01
**Test results (blood)**			
AMH (ng/ml)	1.13 ± 1	4.07 ± 1.09	<0.01
E2 (pg/ml)	138.14 ± 50.57	54.52 ± 25.17	<0.01
CA125 (U/ml)	67.33 ± 28.63	14.43 ± 4.49	<0.01
NLR	2.26 ± 0.8	1.82 ± 0.38	<0.05
**r-ASRM score**			
Stage 1–2	7		
Stage 3–4	9		

*BMI, body mass index, mass (kg)/height (m)/height (m); AMH, anti-Müllerian hormone; E2, estradiol; CA125, carbohydrate antigen 125; NLR, neutrophil-to-lymphocyte ratio; r-ASRM, revise American Society for Reproductive Medicine, score, stage 1 (score 1–5), stage 2 (score 6–15), stage 3 (score 16–40), stage 4 (score > 40).*

### Vaginal Microbiota Difference Between Patients With Endometriosis and Healthy Women

**FIGURE 1:**
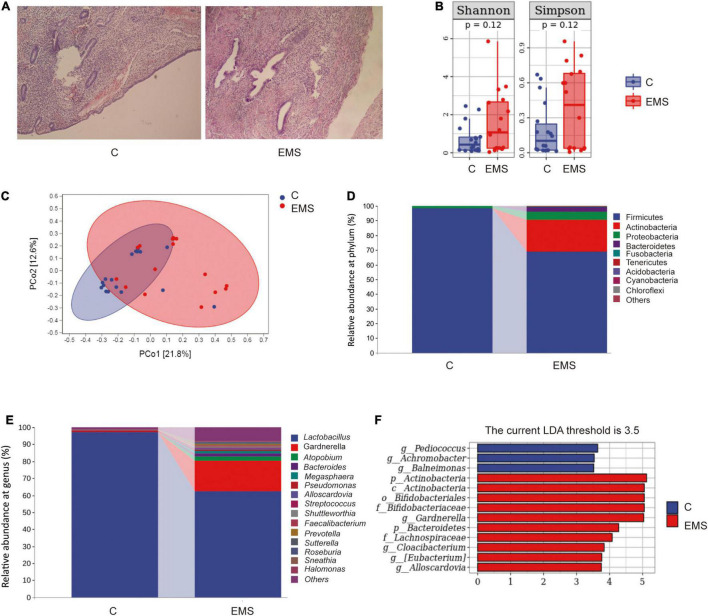
Dysbiosis of vaginal microbiota patterns in patients with endometriosis and control women. **(A)** The typical endometrium pathological examination results of endometriosis and control women. **(B)** Comparison of alpha diversity indices (Chao1 index and Shannon diversity index) between two groups. **(C)** Principal coordinate analysis based on unweighted UniFrac distances revealed that the normal women bacterial communities clustered separately from patients with endometriosis bacterial communities, which were more similar to each other. Each circle represents a single sample, colored by group. The eigenvalues of axe principal coordinate (PC1 and PC2) were 21.8% and 12.6%, respectively. **(D,E)** Taxonomic composition analysis of phylum **(D)** and genus **(E)** between two groups. **(F)** LDA effect size identified the most differentially abundant taxa between the two groups. Only taxa meeting an LDA significant threshold of > 3.5 are shown.

The pathological examination results of the endometriosis group (EMS) and control group (C) were accomplished after the operation. The typical eutopic uterus (left) included endometrium, glands, and stroma, whereas ectopic lesion (right) consisted largely of glands and stroma (Figure 1A). High-throughput sequencing method was used to compare the microbiota between vaginal secretions from endometriosis group and control group (Figures 1B–F). We did not observe significant differences between endometriosis group and control group for Shannon index (estimation total species) and Chao1 index (for community diversity) of alpha diversity, although Shannon index and Chao1 index were higher in endometriosis group than control group (Figure 1B). In addition, according to PCoA, the vagina microbiome of the endometriosis group differed significantly from that of the control group using the unweighted UniFrac distance (Figure 1C). The vaginal bacterial community consisted of 154 genera belonging to 28 phyla in patients with endometriosis and in the healthy control group. At both the phylum and genus levels, the microbial composition of patients with endometriosis differed from that of the control group (Figures 1D,E). The abundance comparisons of predominant genera showed that actinobacteria (especially *Gardnerella* and *Atopobium*) was significantly enriched (from 0.23 to 21.59%), whereas Firmicutes (especially *lactobacillus*) were depleted in patients with endometriosis (from 98.25 to 68.79%). To further identify which bacterial taxa was distinct between the endometriosis and control group, we performed LDA effect size (LEfSe) analysis (LDA 3.5) and identified 13 genera showing significant differences (Figure 1F). These results revealed profound changes in the vaginal microbiome structure of the endometriosis group, which indicates the importance of vaginal microbiota in the development of endometriosis.

### Disorder of Vaginal Microbiota May Promote Endometriotic Lesion Progression by NF-κB Signaling Pathway

**FIGURE 2:**
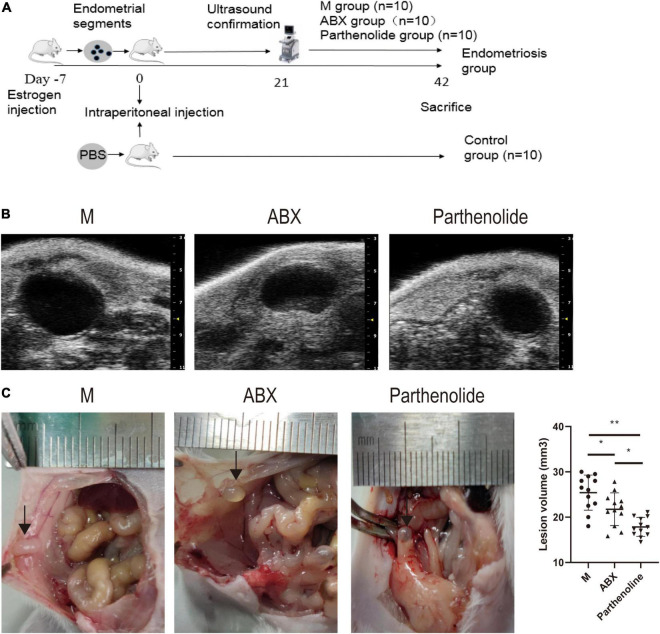
Mixed antibiotics and parthenolide inhibit endometriotic lesion progression. **(A)** Schematic of experimental timeline and procedures. **(B)** High-resolution ultrasound confirmed the change in lesion size with treatment. **(C)** Representative gross images and volumes of ectopic lesions from the indicated treatment groups 3 weeks after established endometriosis confirmed by ultrasound examination. Data are presented as mean ± SE; mock (*n* = 10), ABX (*n* = 10), and parthenolide (*n* = 10). *p < 0.05, **p < 0.01, and ns, non-signficant.

To find out whether vaginal microorganisms affected the progression of endometriosis of established endometriotic lesions confirmed by ultrasound examination, an absorbable gelatin sponge immersed in broad-spectrum antibiotics water was placed in the vagina of mice, while mock mice received absorbable gelatin sponge water alone. Meanwhile, we used parthenolide in another group of mice with established models to compare the therapeutic effects of ABX (Figure 2A). To reduce unnecessary animal death, high-resolution ultrasound was used to confirm the success of endometriotic lesions modeling, instead of killing several model mice to observe the lesions in the abdominal cavity (Figure 2B). After 3 weeks of treatment, mice that consumed ABX and parthenolide had smaller endometriotic lesions than those that consumed vehicle alone (mock mice), and the parthenolide group had smaller lesions compared with the ABX group (Figures 2B,C). These experiments indicated that parthenolide and antibiotic treatment reduced the progression of endometriotic lesions.

**FIGURE 3:**
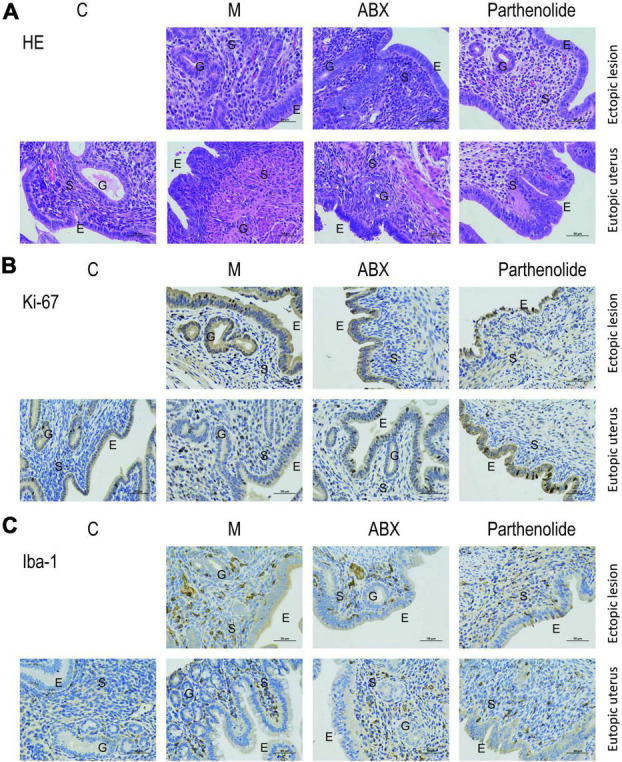
Removing vaginal microorganisms reduces endometriotic lesion progression confirmed by pathological examination. **(A)** Representative hematoxylin and eosin-stained cross-section images of the eutopic uteri and ectopic lesions from the indicated treatment groups. The scale bar (0.5 μm) applies to all images. **(B,C)** Representative cross-sectional images of the eutopic uteri and ectopic lesions immunohistochemistry stained for Ki-67 **(B)** and Iba1 **(C)**.

Hematoxylin–eosin staining and immunohistochemistry techniques were used to uncover the mechanism that affected endometriotic lesion progression. First, we used hematoxylin–eosin staining to demonstrate that ectopic lesions from mock mice had typical endometriosis-like structures, including a thick epithelial layer and glandular areas, whereas lesions from ABX mice and parthenolide mice had thinner epithelial areas and less glands, and this phenomenon was more pronounced in the parthenolide mice (Figure 3A). Additionally, lesions from ABX mice and parthenolide mice had smaller stromal areas than lesions of mock mice. Importantly, the eutopic uteri had similar epithelial, glandular, and stromal areas in both mock groups and two therapeutic groups (Figure 3A). Second, we assessed epithelial proliferation, which is a hallmark of endometriosis in women and is widely used to assess disease progression in rodent models of endometriosis. Consistent with their larger size, lesions from ABX mice and parthenolide mice, especially the parthenolide mice, had significantly less epithelial cells that were positive for the proliferation marker ki-67 than did lesions from mock mice (Figure 3B). Third, we examined macrophage infiltration in lesions because macrophages drive lesion growth and vascularization in the mouse model of endometriosis. Lesions from ABX mice and parthenolide mice, especially the latter, contained significantly less macrophages than did lesions from mock mice (Figure 3C).

**FIGURE 4:**
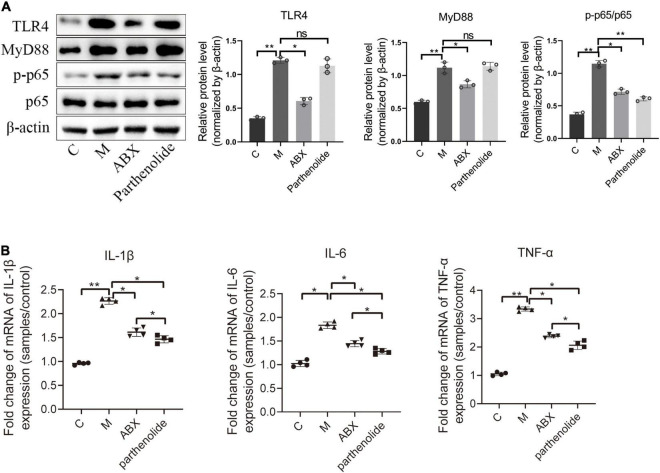
Inflammatory cytokines and key proteins of the NF-κB signaling pathway were inhibited by ABX and parthenolide in EMS mice. **(A)** Western blot analysis of TLR4, MyD88, p65, and p-p65 expression in ectopic lesions, β-actin was used as an internal control (*n* = 3). The relative expressions of TLR4, MyD88, p65 and p-p65 were quantified by ImageJ. **(B)** qPCR-based relative expression of IL-1β, TNF-α, and IL-6 levels in peritoneal fluid from the indicated treatment groups. Data are presented as mean ± SE (*n* = 4). Data are presented as means ± SD. One-way repeated-measures ANOVA with Tukey’s test for multiple comparisons, *p < 0.05, **p < 0.01, and ns, non-significant.

Finally, key proteins in the NF-κB signal transduction pathway were further studied. ABX significantly reduced the expression of TLR4, MyD88, and p65/p-p65, with the parthenolide inhibiting p65/p-p65 (Figure 4A). We also measured peritoneal concentrations of IL-1β, as this cytokine was reported to be elevated in the peritoneal fluid of women with endometriosis. ABX mice and parthenolide mice had less peritoneal IL-1β than mock mice, and the concentration in parthenolide mice was lower than that in ABX mice. Similarly, ABX mice and parthenolide mice had less peritoneal concentrations of TNF-α and IL-6 than mock mice, with a lower concentration in parthenolide mice compared to ABX mice (Figure 4B). Together, these data indicated that vaginal microbiota may regulate endometriotic lesion progression by the NF-κB signaling pathway.

### Vaginal Microbiota From Healthy Mice Inhibits Endometriotic Lesion Progression

**FIGURE 5:**
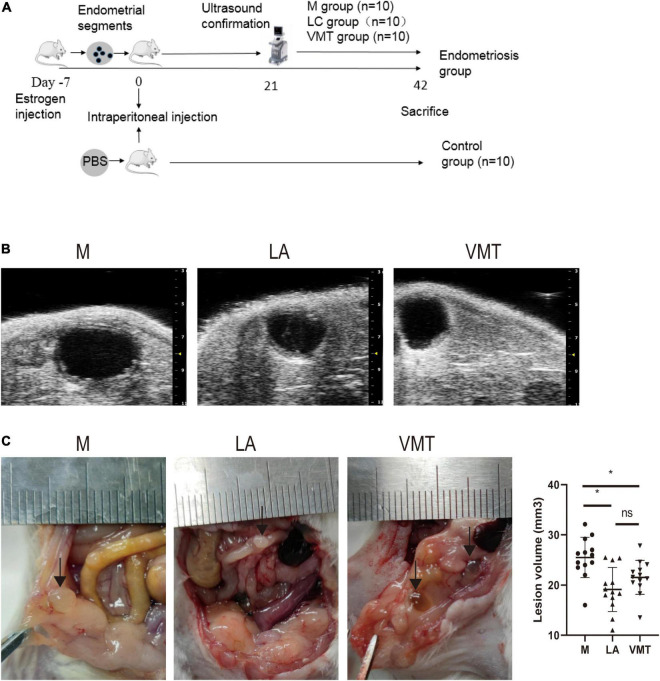
VMT inhibit endometriotic lesion progression. **(A)** Schematic of experimental timeline and procedures. **(B)** High-resolution ultrasound confirmed the change in lesion size with treatment. **(C)** Representative gross images and volumes of ectopic lesions from the indicated treatment groups 3 weeks after established endometriosis confirmed by ultrasound examination. Data are presented as mean ± SE; mock (*n* = 10), ABX (*n* = 10), and parthenolide (*n* = 10). *p < 0.05 and ns, non-signficant.

Considering that the antibiotic-removed vaginal microbiota of endometriosis mice could alleviate endometriosis, we wondered whether vaginal microbiota from healthy mice was sufficient to restrain endometriotic lesion progression of mice with endometriosis. To address this possibility, mice were intraperitoneally injected with fragments or PBS on day 0, and ultrasound was used to confirm the success of modeling on day 21 like the previous experiment. After that, some mock mice were subcutaneously injected LA (as positive control group) whereas others were treated with VMT from days 22 to 42 (Figure 5A). VMT mice transplanted with vaginal secretions from healthy mice and LA mice developed endometriotic lesions that were smaller in volume than those in mock mice (Figures 5B,C).

**FIGURE 6:**
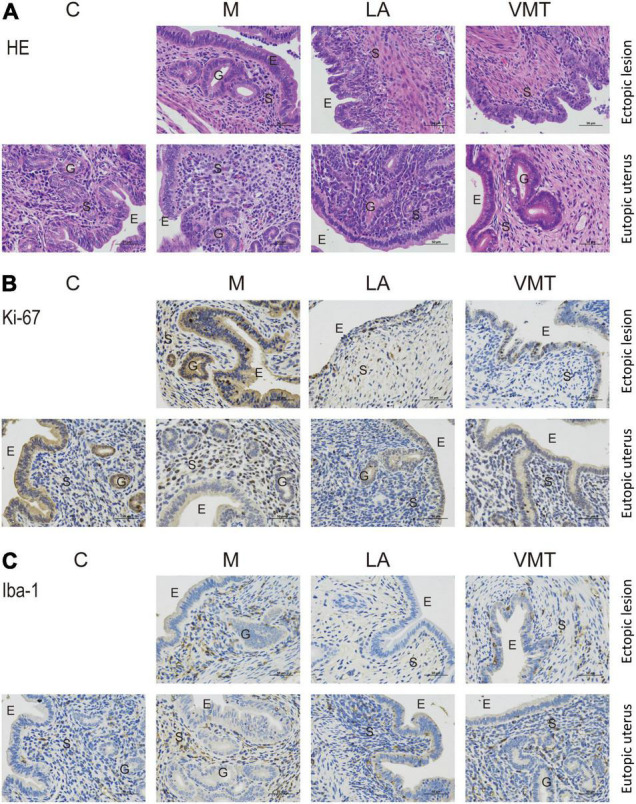
VMT inhibits endometriotic lesion progression confirmed by pathological examination. **(A)** Representative hematoxylin and eosin-stained cross-section images of the eutopic uteri and ectopic lesions from the indicated treatment groups. The scale bar (0.5 μm) applies to all images. **(B,C)** Representative cross-sectional images of the eutopic uteri and ectopic lesions immunohistochemistry stained for Ki-67 **(B)** and Iba1 **(C)**.

We observed typical endometriosis-like histology (the presence of glands and thick epithelial layer) in lesions from mock mice, who stuffed absorbable gelatin sponge immersed in PBS. In contrast, lesions from LA mice and VMT mice lacked glands and had a thinner epithelial layer (Figure 6A). We also detected cell proliferation marker Ki-67 and macrophage marker Iba-1, respectively. Compared with M group, VMT group and LA group showed a decreased expression of the two factors, with no significant difference between VMT and LA groups (Figures 6B,C).

**FIGURE 7:**
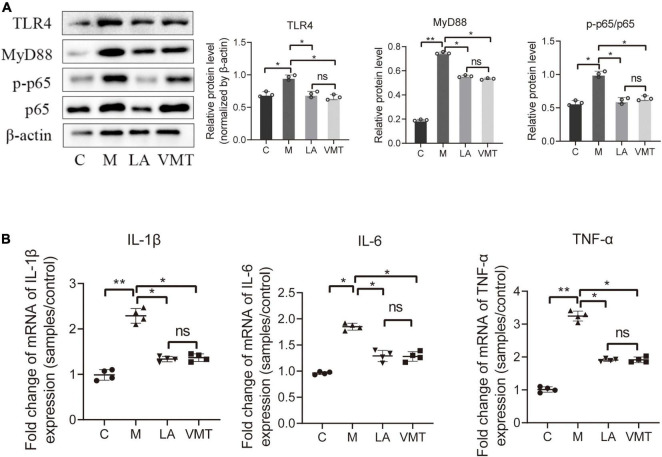
VMT inhibit inflammatory cytokines and key proteins of the NF-κB signaling pathway. **(A)** qPCR-based relative expression of IL-1β, TNF-α, and IL-6 levels in peritoneal fluid from the indicated treatment groups. Data are presented as mean ± SE (*n* = 4). **(B)** Western blot analysis of TLR4, MyD88, p65, and p-p65 expression in ectopic lesions, β-actin was used as an internal control (*n* = 3). The relative expressions of TLR4, MyD88, p65, and p-p65 were quantified by ImageJ. Data are presented as means ± SD. One-way repeated-measures ANOVA with Tukey’s test for multiple comparisons, *p < 0.05, **p < 0.01, and ns, non-significant.

Leuprorelin acetate mice and VMT mice contained less IL-1β, IL-6, and TNF-α in the peritoneal fluid than in mock mice (Figure 7A). In addition, TLR4, MyD88, and p65/p-p65 (NF-κB signaling pathway) in ectopic lesions of LA mice and VMT mice were downregulated (Figure 7B). According to the above results, it could be concluded that vaginal microorganisms may inhibit in the progression of endometriosis.

## Discussion

Endometriosis is a chronic, multisystem inflammatory disease mainly manifested by endometrial cells that colonize and grow outside the uterine cavity, which affects about 10% of women of childbearing age and seriously endangers women’s physical and mental health. Although many hypotheses, such as retrograde menstruation, endometrial stem cell implantation, müllerian remnant abnormalities, and coelomic metaplasia, have been put forward to elucidate the pathogenesis of endometriosis, but they could not fully explain the progression of all types of endometriotic lesions. In addition, its definite diagnosis and treatment are often traumatic for requiring surgery to remove lesions. This study explored the relationship between endometriosis and vaginal microorganisms and studied the effects of VMT in endometriosis as a natural orifice transluminal method, which may improve the current treatment dilemma.

The relationship between vaginal microorganisms and endometriosis is not fully understood. This study compared the differences in vaginal microbiota between endometriosis women and healthy women by high-throughput sequencing. Similar to the report from Chen, the alpha diversity in vagina secretion from the endometriosis group and healthy women group did not show significant differences, but the microbial constituent was significantly altered (22). The patients had a higher relative abundance of actinobacteria (especially *Gardnerella* and *Atopobium*) and a lower abundance of Firmicutes (especially *Lactobacillus*) compared with healthy women. These results are coordinated with the study of Ata et al., which reported the vaginal, cervical, and gut microbiota between women with and without endometriosis (31). Wei et al. also reported the reduction of the vaginal *Lactobacillus genus* (23). Interestingly, patients with BV have similar vaginal microbiota changes, including a decreased abundance of *Lactobacillus genus* and an increased abundance of anaerobic bacteria, such as *Gardnerella genus* and *Atopobium genus* (32, 33).

*Lactobacillus genus* is a dominant microbiota to maintain the microecological balance of the vagina. In addition, to keep the acidic environment of the vagina, *Lactobacillus* can also secrete bacteriocin and other anti-microbiological factors to inhibit or kill pathogenic microorganisms (34). At the same time, it can prevent the pathogenic microorganisms from extending to the epithelial cells of the vagina through the competitive rejection mechanism. *Atopobium genus* was reported as detrimental bacteria related to obstetric bacteremia, BV, and endometrial cancer (35–37). *Gardnerella genus* overgrow in plenty diseases, such as BV, in which has a higher virulence potential and ability to adhere to epithelium than twenty-nine other BV-associated bacteria (38). Although we observed the significant increase of these two harmful bacteria and the decrease of beneficial bacteria, such as *Lactobacillus*, the detailed mechanism of these bacteria in the onset of endometriosis remained to be further studied.

Considering the changed microbiota in our clinical study, the intestinal microbiota of endometriosis mice reported by the previous study, and the function of antibiotics, the effects of antibiotics in treating endometriosis were studied compared with another drug, such as parthenolide (19, 39). The results revealed that the mixed antibiotics could reduce the volume of ectopic lesions in female mice with endometriosis. Also, inflammatory cytokines IL-1β, IL-6, and TNF-α were decreased. Some key proteins of TLR4, MyD88, and p65/p-p65 in the NF-κB signaling pathway were downregulated. Chadchan et al. reported the inhibitory effect of intestinal antibiotics on female mice with endometriosis, which was similar to our results, but it did not further study the signaling pathway (40). Takai et al. reported similar therapeutic effects and detailed mechanisms of NF-κB signaling pathway inhibitors (parthenolide inhibits NF-κB signaling pathway by suppressing both the phosphorylation of IκB kinase complex and IκBα degradation) for endometriosis mice, including inhibition effect for number, the weight of endometriotic lesions, the level of vascular endothelial growth factor (*VEGF*), monocyte chemotactic protein-1 (*MCP-1*), and leukemia inhibitory factor (*LIF*) gene expression, which established the basis for our experimental comparison (27).

The removal of vaginal microorganisms by antibiotics from mice with endometriosis could inhibit the progress of this disease, and thus, it is reasonable to hypothesis that the restoration of normal vaginal microecology has an advantageous impact on endometriotic lesions. VMT, a promising technique that grafting the whole vaginal microbiota from a healthy donor to the receptor with vaginal microbiota dysbiosis, has been studied in various gynecological diseases and has been applied to treat intractable BV of rats and humans (30, 41, 42). LA, one of the gonadotropin-releasing hormone analogs, is the first-line medicine for the clinical treatment of endometriosis. LA could inhibit the progression of ectopic lesions by reducing estrogen levels, which is essential for ectopic lesion growth. Although LA has a satisfactory therapeutic effect in endometriosis, it needs to be used many times and is usually expensive. What is worse, there are various adverse reactions, such as hot flashes, vaginal dryness, decreased libido, and osteoporosis. We compared the therapeutic effects and found that VMT had almost consistent inhibitory ectopic lesion progression effect in mice like LA, along with inflammatory cytokines reduced and key proteins of NF-κB signaling pathway downregulated, suggesting that vaginal bacteria play an important role in endometriotic lesion progression and reconstructing the vaginal microbiota microenvironment to normal could effectively reduce the inflammatory level of endometriosis mice.

After the ectopic lesion was implanted successfully, proinflammatory cytokines were elevated in the peritoneal fluid (43). In turn, inflammatory factors, for example, IL-1β, also promoted the growth of lesions (44, 45). Meanwhile, vasculogenesis and VEGF from immune cells mediated ectopic lesion progression, and the macrophages particularly played an important role in it (46). Our findings showed that endometriosis mice treated with ABX or VMT had fewer macrophages, less endometriotic cell proliferation in their lesions, and lower peritoneal inflammatory cytokines concentration than mock mice. Moreover, pelvic inflammatory responses could result from vaginal bacteria. Therefore, it is speculated from our results that disordered vaginal microbiota may promote the progression of endometriosis, but its detailed mechanism needs to be further explored in the future.

Several limitations in this study need to be improved in the future. (i) The number of women included in the study was small, which affects the number of patients in each stage of r-ASRM. We can further analyze the patients in different stages if the number of samples increases. For example, vaginal microorganisms have been used to predict the stage of endometriosis women (20). (ii) There may be other mechanisms to inhibit the progression of endometriotic lesions except the NF-κB signaling pathway. For example, LA not only inhibits the level of estrogen, but also restrains the inflammatory level of endometriosis (47). So, we infer that there may be complicated mechanisms form the combined action. (iii) There are differences in vaginal microbial genus between female mice and women. The vaginal microbiota of female mice is mainly *Enterobacteriaceae, Streptococcus* spp., *Staphylococcus* spp., *Corynebacterium* spp., and *Lactobacillus* spp., whereas women of childbearing age is mainly *Lactobacillus* of Firmicutes (48). Whether the results of the animal experiment could be directly applied to women of childbearing age needs research in the future after the adoption of the ethics document.

In conclusion, our results show that vaginal bacteria can promote the progression of endometriosis and VMT shows great benefits on it. It should be further studied and has the potential to be applied in the clinic, which may be a new treatment strategy for endometriosis and other gynecological diseases.

## Data Availability Statement

The datasets presented in this study can be found in online repositories. The names of the repository/repositories and accession number(s) can be found in the article/supplementary material.

## Ethics Statement

The studies involving human participants were reviewed and approved by the Ethical Committee of the Second Affiliated Hospital of Nanchang University. The patients/participants provided their written informed consent to participate in this study. The animal study was reviewed and approved by the Tab of Animal Experimental Ethical Inspection of Nanchang Royo Biotech Co., Ltd.

## Author Contributions

BT and TC designed the current experiments. FL performed the experiments. FL, YZ, YF, BM, YX, and KW analyzed all data and wrote the manuscript. All authors contributed to the article and approved the submitted version.

## Conflict of Interest

The authors declare that the research was conducted in the absence of any commercial or financial relationships that could be construed as a potential conflict of interest.

## Publisher’s Note

All claims expressed in this article are solely those of the authors and do not necessarily represent those of their affiliated organizations, or those of the publisher, the editors and the reviewers. Any product that may be evaluated in this article, or claim that may be made by its manufacturer, is not guaranteed or endorsed by the publisher.
